# A commentary on “Usefulness of fluorescence imaging with indocyanine green for evaluation of bowel perfusion in the urgency setting: a systematic review and meta-analysis”

**DOI:** 10.1097/JS9.0000000000002970

**Published:** 2025-07-19

**Authors:** Xinchao Yang, Liang Chen, Xionghui He

**Affiliations:** a Department of Cardiothoracic Surgery, Hainan General Hospital (Hainan Affiliated Hospital of Hainan Medical University) Haikou, Hainan Province, China; b Department of General Surgery, Hainan General Hospital (Hainan Affiliated Hospital of Hainan Medical University) Haikou, Hainan Province, China

*Dear Editor,*

Indocyanine green fluorescence imaging (ICGFI) has been widely used to assessing anastomotic perfusion in elective gastrointestinal surgery, particularly in the context of rectal cancer surgery^[1]^ and esophageal cancer surgery^[2]^. However, the safety and effectiveness of this approach in emergency settings remain unclear. To address this issue, Rizzo *et al*^[3]^, conducted a meta-analysis by incorporating all eligible studies up to 1 July 2023. In their meta-analysis, the authors included a total of six studies including 297 patients and demonstrated that ICGFI had a comparable reoperation rate compared to the control group. The authors should be commended for their efforts in confirming the feasibility of ICGFI in evaluating bowel perfusion in those patients. Nevertheless, there are still some concerns that need clarification to further enhance the quality of this study.

Firstly, when interpreting the forest plot (in Figure 2 of their study), Rizzo *et al*^[3]^ emphasized that although the risk difference (−0.04 [95% CI: −0.147-0.060]) was comparable between the ICGFI and control groups, the pooled result demonstrated statistical significance with a *P* value of 0.029. However, this *P* value (0.029) reflects significant heterogeneity (*I*^2^ = 59.9%) rather than confirming the statistical significance of the pooled result. Therefore, we strongly urge the authors to thoroughly rectify all relevant misrepresentations.

Secondly, we are curious why the authors only compared the difference in reoperation rates between the two groups. Obviously, the single measure of reoperation rate does not support their conclusion that ICGFI is a safe and feasible technique. In fact, one of the primary advantages of ICGFI lies in its ability to prevent anastomotic leakage. Therefore, we strongly recommend the authors to provide relevant comparative results regarding the incidence of anastomotic leakage between both groups. Additionally, it would be highly beneficial if the authors could include comparisons for other surgical outcomes such as operative time, intraoperative blood loss, postoperative complications, and mortality.

Thirdly, there is an embarrassing error in the abstract of this article, where the references were inserted improperly within the results section. We strongly advise authors to thoroughly review their manuscripts prior to submission and emphasize the importance of conscientiousness and responsibility from reviewers.

This study is compliant with the TITAN Guidelines 2025^[4]^.

## Data Availability

No data used in this letter to the editor.

## Research registration unique identifying number (UIN)

No need for this letter to the editor.
